# A first-in-human phase 1 trial to evaluate the safety and immunogenicity of the candidate tuberculosis vaccine MVA85A-IMX313, administered to BCG-vaccinated adults

**DOI:** 10.1016/j.vaccine.2016.01.062

**Published:** 2016-03-08

**Authors:** Alice Minhinnick, Iman Satti, Stephanie Harris, Morven Wilkie, Sharon Sheehan, Lisa Stockdale, Zita-Rose Manjaly Thomas, Raquel Lopez-Ramon, Ian Poulton, Alison Lawrie, Samantha Vermaak, Alexandre Le Vert, Judith Del Campo, Fergal Hill, Paul Moss, Helen McShane

**Affiliations:** a The Jenner Institute, University of Oxford, Oxford OX3 7DQ, UK; b School of Cancer Sciences, University of Birmingham, Edgbaston, Birmingham B15 2TT, UK; c IMAXIO, 99, rue de Gerland, 69007 Lyon, France

**Keywords:** Tuberculosis, Vaccine, IMX313, MVA85A, Immunogenicity

## Abstract

**Introduction:**

There is an urgent need for a new and effective tuberculosis vaccine because BCG does not sufficiently prevent pulmonary disease. IMX313 is a novel carrier protein designed to improve cellular and humoral immunity. MVA85A-IMX313 is a novel vaccine candidate designed to boost immunity primed by bacillus Calmette-Guérin (BCG) that has been immunogenic in pre-clinical studies. This is the first evaluation of IMX313 delivered as MVA85A-IMX313 in humans.

**Methods:**

In this phase 1, open-label first-in-human trial, 30 healthy previously BCG-vaccinated adults were enrolled into three treatment groups and vaccinated with low dose MVA85A-IMX313 (group A), standard dose MVA85A-IMX313 (group B), or MVA85A (group C). Volunteers were followed up for 6 months for safety and immunogenicity assessment.

**Results:**

The majority of adverse events were mild and there were no vaccine-related serious AEs. Both MVA85A-IMX313 and MVA85A induced a significant increase in IFN-*γ* ELISpot responses. There were no significant differences between the Ag85A ELISpot and intracellular cytokine responses between the two study groups B (MVA85A-IMX313) and C (MVA85A) at any time point post-vaccination.

**Conclusion:**

MVA85A-IMX313 was well tolerated and immunogenic. There was no significant difference in the number of vaccine-related, local or systemic adverse reactions between MVA85A and MVA85A-IMX313 groups. The *mycobacteria*-specific cellular immune responses induced by MVA85A-IMX313 were not significantly different to those detected in the MVA85A group. In light of this encouraging safety data, further work to improve the potency of molecular adjuvants like IMX313 is merited.

This trial was registered on clinicatrials.gov ref. NCT01879163.

## 1 Introduction

The lack of a safe and effective tuberculosis (TB) vaccine is a public health emergency. TB causes a major global health burden, with an estimated 9.0 million incident cases and 1.5 million deaths each year [1]. In addition, the emergence of drug resistant forms of TB further magnifies the difficulty of TB control. The only currently available licensed vaccine is *Mycobacterium bovis* (*M. bovis*) bacillus Calmette-Guérin (BCG). BCG prevents disseminated disease in childhood [2], [3], but does not provide sufficient or consistent protection against pulmonary TB [3], [4], [5]. It confers varying effectiveness across different populations for reasons that are not well understood. An improved TB vaccine is urgently required.

Technologies that increase vaccine immunogenicity and effectiveness are critical to achieving this goal. One example is the immunogenicity-enhancing protein technology, IMX313. IMX313 is a small protein domain that self-assembles into a nanoparticle with seven identical chains. The 55 amino acid sequence is a hybrid of the oligomerisation domains of two chicken C4b-binding proteins, both distant homologues of human Complement 4 binding protein (C4bp) [6]. In pre-clinical studies, IMX313 has an adjuvant-like effect when fused with protein antigens [6]. Here we assess the safety and immunogenicity of IMX313 with a clinically advanced candidate TB vaccine – Modified Vaccinia virus Ankara expressing the immunodominant *Mycobacterium.tuberculosis (M.tb)* antigen 85A, MVA85A.

MVA85A was designed to boost BCG induced protection and is the only TB subunit vaccine to be evaluated in an efficacy trial. In phase I trials, MVA85A was highly immunogenic and induced potent Ag85A specific CD4+ T-cell responses in BCG-vaccinated adults [7], [8], [9]. Despite this, in a South African phase IIb trial in 2797 South African, BCG-vaccinated infants, MVA85A was safe, but did not improve protective efficacy above the level achieved by BCG alone [10]. The reasons for this could be manifold, but one hypothesis is that MVA85A elicited insufficient IFN-*γ*-producing T helper (Th1) and/or IL-17-producing CD4+ (Th17) cell responses. After vaccination with MVA85A there was only a modest induction of Th1 and Th17 antigen specific T-cell responses, which was 10-fold lower than that seen in UK adults [9].

Efforts to improve the immunogenicity of MVA85A are ongoing. In one strategy, MVA85A has been combined with IMX313. In both mice and non-human primate studies IMX313 improved the immunogenicity of MVA85A [11]. Here we describe the first clinical evaluation of IMX313, administered as MVA85A-IMX313, and compared the safety and immunogenicity profile with MVA85A in a phase I trial in BCG-vaccinated UK adults.

## 2 Methods

### 2.1 Study design

We undertook a phase I, randomised, open-label, first-in-human clinical trial in 30 BCG-vaccinated adults to assess the safety and immunogenicity of a candidate TB vaccine, MVA85A-IMX313.

We enrolled volunteers following their written informed consent under a protocol approved by the UK Medicines and Healthcare products Regulatory Agency (EudraCT 2013-000678-31) and the NRES South Central – Oxford Research Ethics Committee (ref. 13/SC/0207). Recruitment took place at the Centre for Clinical Vaccinology and Tropical Medicine, Oxford, and the NIHR Wellcome Trust Clinical Research Facility, Birmingham. This trial was registered on clinicatrials.gov (ref. NCT01879163) and was conducted according to the principles of the Declaration of Helsinki and Good Clinical Practice.

### 2.2 Participants

We enrolled volunteers from the general population around Oxford and Birmingham. Volunteers were healthy, aged between 18 and 55 and had received BCG at least 6 months prior to their date of enrolment. They had normal baseline haematology and biochemistry and were hepatitis B, C and HIV negative. Latent *M.tb* infection was excluded by a negative ex vivo IFN-*γ* ELISpot response to *M.tb* early secreted antigenic target 6 kDa (ESAT6) and the 10-kDa culture filtrate protein (CFP10) peptides. The full inclusion and exclusion criteria are described in Supplementary Methods 1.

### 2.3 Vaccines

Clinical-grade MVA85A (lot number 0050811) was constructed as previously described [12]. MVA85A-IMX313 (lot number 0010812), which expresses the IMX313 fusion protein, was constructed as described elsewhere [11]. Both vaccines were produced under Good Manufacturing Practice conditions (GMP) by IDT Biologika GmbH, Germany (IDT).

### 2.4 Clinical procedures

The first 6 volunteers were assigned to the starter group (group A), who were administered a low dose of MVA85A-IMX313 (1 × 10^7^ pfu) delivered intradermally in a volume of 150 μL into the upper arm (all injections were administered by a 29G diameter, 12.7 mm length needle). These group A (low dose MVA85A-IMX313) vaccinations occurred step-wise in order to assess safety. The safety of the first volunteer was assessed and 48 h passed before the next two volunteers in group A were vaccinated. The remaining volunteers in group A were vaccinated once the Chief Investigator decided it was safe to proceed. Once all 6 volunteers in group A had been followed up for 14 days, the dose was escalated to 5 × 10^7^ pfu. One volunteer (the first group B volunteer) was assigned to receive the higher dose MVA85A-IMX313 (5 × 10^7^ pfu, delivered intradermally in a volume of 76 μL) and 48 h after vaccination their safety was reviewed before we proceeded to randomisation of the remaining 23 volunteers.

We randomly allocated the remaining 23 eligible volunteers (1:1) to receive intradermal MVA85A-IMX313, 5 × 10^7^ pfu (Group B) or intradermal MVA85A, 5 × 10^7^ pfu, delivered in a volume of 60 μL (Group C). Randomisation was done with sequentially numbered, opaque, sealed envelopes, prepared by an independent statistician, opened by the study clinician at enrolment. Volunteers and laboratory staff were blinded to intervention assignment.

Following vaccination and safety reviews at 30 and 60 min, all volunteers were followed up for a period of 6 months, with clinic visits at days 2, 7, 14, 28, 84 and 168. Volunteers completed diary-cards for the recording of adverse events (AEs) for 7 days post-vaccination. Symptoms were reviewed at each clinic visit, and vaccination site observations (redness, swelling) and vital signs (blood pressure, heart rate, oral or tympanic temperature) were recorded. Safety bloods (full blood count, urea and electrolytes, liver enzymes) were collected on D7 and D84 post-vaccination. Solicited (local injection site: pain, redness, swelling, warmth, itch, scaling; and systemic: documented fever, feverishness, malaise, arthralgia, headache, myalgia, nausea/vomiting, fatigue) and unsolicited AEs were recorded in line-listings for later analysis. Assignment of a causal relationship for AEs was conducted according to predefined criteria specified in the protocol. Blood for immunological assessment was taken at all follow-up visits and peripheral blood mononuclear cells (PBMC) and serum were isolated and cryopreserved.

### 2.5 Immunological evaluations

#### 2.5.1 Ex vivo IFN*γ* Enzyme-Linked ImmunoSpot (ELISpot) assay

ELISpot assays were performed on freshly isolated PBMC, from all volunteers at screening or on day of vaccination and on days 7, 14, 28, 84 and 168 post-vaccination, as previously described [13]. Ag85A single pool of 66-peptides (Peptide Protein Research (PPR), UK), IMX313 peptides and C4bp (IMAXIO, France) were all used at a final concentration of 2 μg/ml. Purified protein derivative (PPD), (Statens Serum Institute, Denmark) was used at 20 μg/ml.

ELISpot plates (Millipore) were coated with capture mAb then incubated overnight at 4 °C. 3 × 10^5^ PBMC were incubated for 18–20 h at 37 °C/5% CO_2_ with Ag85A (single pool of 66 15mer peptides, overlapping by 10 amino acids) (PPR, UK), purified protein derivative (PPD) (Staten Serum Institute, Denmark), MVA CD4 and CD8T cell epitopes (PPR, UK), IMX313 (IMAXIO, France) and hC4bp (provided by Anna Blom, Sweden). Staphylococcal enterotoxin B (Sigma) was used as a positive control and unstimulated PBMC were used as negative controls. Antigen-specific background subtracted results are presented as spot-forming cells (SFC) per million PBMC.

#### 2.5.2 Whole Blood (WB) Intracellular Cytokine Staining (ICS)

Ag85A-specific intracellular cytokines were measured in WB samples as previously described [14]. Blood samples were stimulated with Ag85A peptides, or SEB (Sigma–Aldrich), or left without stimulation as a negative control. αCD28 and αCD49d (BD) were used as co-stimulatory antibodies and samples were incubated at 37 °C/5% CO_2_ for 6 h. Following this, Brefeldin A (Sigma–Aldrich) was added before a further 6 h incubation. Samples were then treated with 2 mM EDTA (GIBCO). Red blood cells were lysed using FACS Lysing solution (BD) and samples were frozen for batched ICS analysis.

Stimulated and fixed WB samples were permeabilised and incubated with antibodies against: CD3 (AF700), IFN-*γ* (PE-Cy7) (Ebioscience), CD4 (Pacific Blue), TNF-*α* (APC), IL-17 (AF488) (Biolegend), CD14 and CD19 (ECD), IL-2 (PE) (Beckman Coulter) and CD8 (APC-H7) (BD). Samples were acquired on LSRII (Becton Dickinson). Cytokines were measured in singlet CD14−CD19−CD3+, CD4+ or CD8+ T cells. Results are presented as percentages of antigen-specific cells producing cytokines minus responses measured in unstimulated cells. Data was analysed using FlowJo (Tree Star Inc., USA), and polyfunctional cytokine immune responses were analysed using Spice software (http://exon.niaid.nih.gov/spice/).

#### 2.5.3 Antibody Enzyme Linked Immunosorbent assay (ELISA)

Levels of Immunoglobulin G (IgG) were measured in serum samples collected on days 0, 14 and 28 as previously described [15]. Insert-specific IgG responses were measured to recombinant Ag85A (Lionex, Germany). Anti-vector IgG responses were measured to wild-type MVA (Vector Core Facility, Jenner Institute, Oxford), IMX313 (IMAXIO, France) and hC4bp proteins (provided by Anna Blom, Sweden). Results are presented as fold change in antigen-specific response of IgG (Optical Density OD) from the value at D0.

IgG subclasses levels to 85A or IMX313 antigen were determined in the sera by an indirect plate ELISA. The wells of ELISA plates (Nunc) were sensitised with Ag85A, IMX313 and hC4bp proteins (100 μL/well in carbonate buffer pH 9.6 at 4 °C, overnight), followed by blocking with 10% bovine foetal serum (non-USA origin, sterile-filtered, Sigma) in PBS (pH 7.4) for 2 h at room temperature. Plates were washed twice with PBS containing 0.05% Tween 20 (PBS/T), followed by addition of 100 μL/well of optimally diluted sera (1/50), in duplicate wells. Plates were incubated at 37 °C for 1 h, then washed three times with PBS/T. After washing, 100 μL of optimally diluted (1/1000) anti-human IgG peroxidase conjugate (anti-human IgG1, anti-human IgG2, anti-human IgG3, anti-human IgG4, Fisher Scientific) in FBS-PBS was added to each well. Plates were incubated for 1 h at 37 °C, then washed five times with PBS/T before the addition of 100 μL per well of TMB HRP Substrate for ELISA (UP664780, Interchim). The reaction was stopped after 20 min with 2 N H_2_SO_4_, and OD values were read at 405 nm. Results are presented as fold change in OD from the value at Day 0.

All serum samples from each volunteer were run on the same ELISA plate to control for assay variability. A pool of Ag85A-positive sera was included in all plates.

### 2.6 Statistical analysis

All volunteers were randomised and vaccinated according to protocol, and so our analyses were per protocol.

The primary study outcome was safety as assessed by the frequency and severity of vaccine-related local and systemic AEs. Safety data was summarised by frequency and severity of AEs using descriptive statistics. Statistical analyses to compare the standard dose groups were performed using GraphPad Prism. The Mann–Whitney *U*-test was used to determine differences between groups.

The secondary study outcome was immunogenicity. ELISpot, WB ICS and antibody response statistical analyses were performed using GraphPad Prism. The Mann–Whitney *U*-test and unpaired *t*-test were used to determine differences between groups. The Wilcoxon matched pairs signed rank test was used to detect differences between time points in the same group. Area Under the curve (AUC) was used to examine overall responses during follow-up period.

## 3 Results

**Fig. 1:**
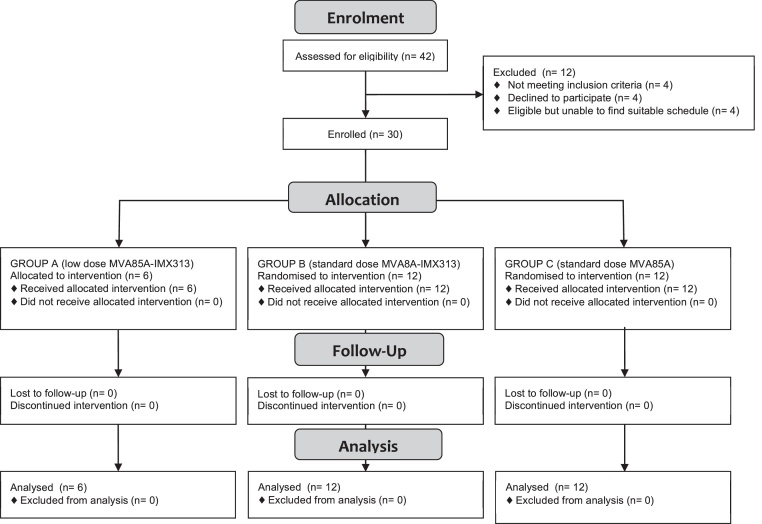
Study profile. CONSORT flow diagram showing volunteer recruitment and follow up. Group A (1 × 10^7^ pfu intradermal MVA85A-IMX313) was fully enrolled first. Once safety data was reviewed, one volunteer was then allocated to group B (5 × 10^7^ pfu intradermal MVA85A-IMX313). Following another planned pause for a review of safety data, the remaining 23 volunteers were randomised 1:1 to groups B and C (5 × 10^7^ pfu intradermal MVA85A).

Between July 17 2013 and July 17 2014, 30 of the 42 volunteers screened for eligibility were enrolled in the trial (Fig. 1). The baseline demographics were similar between groups (Table 1).

### 3.1 Vaccine safety

Group A (the low dose MVA85A-IMX313 safety group) proceeded to completion without safety concerns. The numbers of AEs considered at least possibly related to vaccination were broadly similar between groups with medians of 10 in group A (low dose MVA85A-IMX313), 8.5 in group B (MVA85A-IMX313) and 9 in group C (MVA85A). There was no statistical difference between group B and C (*p* = 0.5024). All related AEs and their severities are shown in Table 2. There were no documented temperature rises following vaccination in any group.

**Fig. 2:**
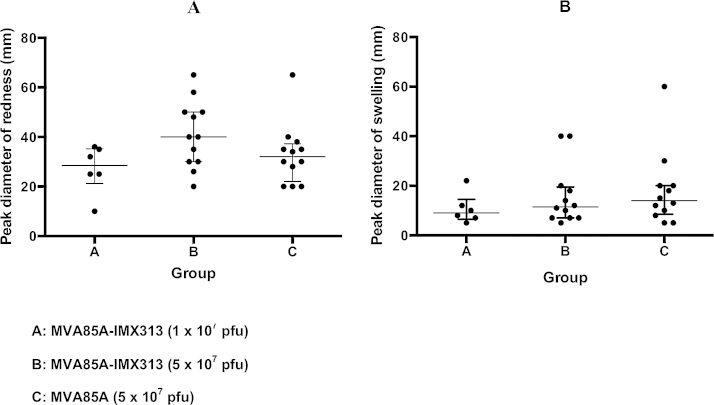
Local reactogenicity. Maximum diameter of redness (A) and swelling (B) at challenge site with median and IQR (dots represent individual volunteers). Medians were compared with the Mann–Whitney *U*-test. The difference in median diameter was not statistically significant between groups (*p* > 0.05).

The majority of AEs were mild in nature. There was no significant difference in the local injection site reaction between groups, with median diameters of redness and swelling of 28.5 mm and 9 mm in group A (low dose MVA85A-IMX313), 40 mm and 11.5 mm in group B (MVA-IMX313) and 32 mm and 14 mm in group C (MVA85A). There was no statistical difference between group B and group C with *p* values of 0.1236 (redness) and 0.6430 (swelling) (Fig. 2). There were four severe AEs that were considered possibly related to vaccination (3 in a group B, MVA85A-IMX313 volunteer and 1 in a group C, MVA85A volunteer). The group B (MVA85A-IMX313) volunteer reported severe nausea from D2 to D3, severe malaise from D2 to D4 and severe fatigue from D2 to D4 following vaccination. The volunteer from group C (MVA85A) had vaccination site swelling that met the criteria for a severe AE that peaked at 60 mm diameter on D4 post-vaccination.

There was one laboratory AE considered related to vaccination. A group C (MVA85A) volunteer had a mildly raised alanine transferase at screening and D7 post-vaccination, which was moderately elevated at D28, mildly elevated at D84 and normalised by D168 post-vaccination. The volunteer was asymptomatic and the AE was considered possibly related to vaccination.

There was one unrelated serious AE reported in the study in a group B (MVA85A-IMX313) volunteer who was admitted overnight to hospital for investigation of chest pain 157 days after vaccination with MVA85A-IMX313. There were no other serious AEs.

### 3.2 Vaccine immunogenicity

#### 3.2.1 MVA85A and MVA85A-IMX313 induces mycobacteria-specific ex vivo IFN-*γ* ELISpot responses

At the low dose (10^7^ pfu MVA85A-IMX313) the vaccine induced significant Ag85A-specific IFN-*γ* ELISpot responses that peaked at D7 post-vaccination (median = 384 SFC/10^6^ PBMC) and remained significant up to D84 post-vaccination. Significantly increased PPD IFN-*γ* responses (median = 447 SFC/10^6^ PBMC) were detected at D7 post-vaccination (data not shown).

**Fig. 3:**
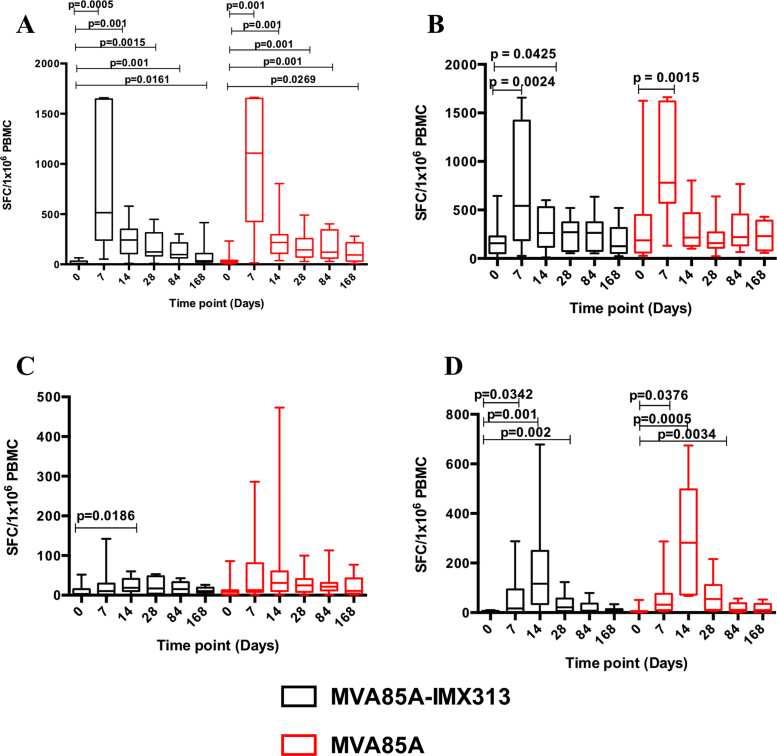
Ex vivo PBMC IFN-*γ* ELISpot responses to Ag85A pool of 66 peptides (A), PPD (B), MVA peptides for CD4 (C), and CD8 epitopes (D) in BCG-vaccinated healthy UK adults boosted with either MVA85A-IMX313 or MVA85A (5 × 10^7^ pfu). Box and whisker plots show median, inter-quartile range, minimum and maximum values. The Wilcoxon matched pairs signed rank test was used to detect differences between time points in the same group and *p*-values have not been adjusted for multiple comparisons. There were no significant differences in the overall antigen-specific IFN-*γ* responses between the two study groups (Mann–Whitney test for AUC, *p* = 0.5059, *p* = 0.5059, *p* = 0.2721 and *p* = 0.1585 for Ag85A-, PPD-, MVA (CD4)- or MVA (CD8)-specific responses respectively).

Vaccination of BCG-primed UK adults with MVA85A-IMX313 or MVA85A, both at 5 × 10^7^ pfu, induced a significant increase in Ag85A-specific IFN-*γ* responses that was durable up to 6 months following vaccination. These responses peaked at D7 post-vaccination with a median IFN-*γ* response of 515 SFC/10^6^ PBMC in the MVA85A-IMX313 group compared to 1107.5 SFC/10^6^ PBMC in the MVA85A group (Fig. 3A). There were no significant differences between the Ag85A ELISpot responses in the two groups B (MVA85A-IMX313) and C (MVA85A) at any time point post-vaccination, or in the overall Ag85A-specific IFN-*γ* response (AUC, *p* = 0.5059).

There was a significant increase in PPD-specific IFN-*γ* ELISpot responses in both groups at D7 in comparison to baseline with a median IFN***-**γ* response of 542.5 SFC/10^6^ PBMC in group B (MVA85A-IMX313) and 780 SFC/10^6^ PBMC in group C (MVA85A) (Fig. 3B). The PPD response was not significantly different between the MVA85A-IMX313 and MVA85A groups (AUC, *p* = 0.5059).

ELISpot IFN-*γ* responses to MVA CD4+ T cell epitopes peaked at D14 post-vaccination, with median responses of 19 SFC/10^6^ PBMC in group B (MVA85A-IMX313) and 31 SFC/10^6^ PBMC in group C (MVA85A) (Fig. 3C). ELISpot responses to MVA CD8+ T cell epitopes were significantly induced in both groups and these responses also peaked at D14 with a median response of 117 SFC/10^6^ PBMC in group B (MVA85A-IMX313) and 282 SFC/10^6^ PBMC in group C (MVA85A), remaining significantly increased until D28. There were no significant differences in responses to MVA epitopes between the two study groups (AUC, *p* = 0.2721 and *p* = 0.1585 for MVA CD4+ T cells epitopes and MVA CD8+ T cells epitopes respectively) (Fig. 3D).

Vaccination with MVA85A-IMX313 or MVA85A alone did not induce ELISpot responses to IMX313 or C4bp peptides (Supplementary Material 1).

#### 3.2.2 Total whole blood intracellular cytokine response

**Fig. 4:**
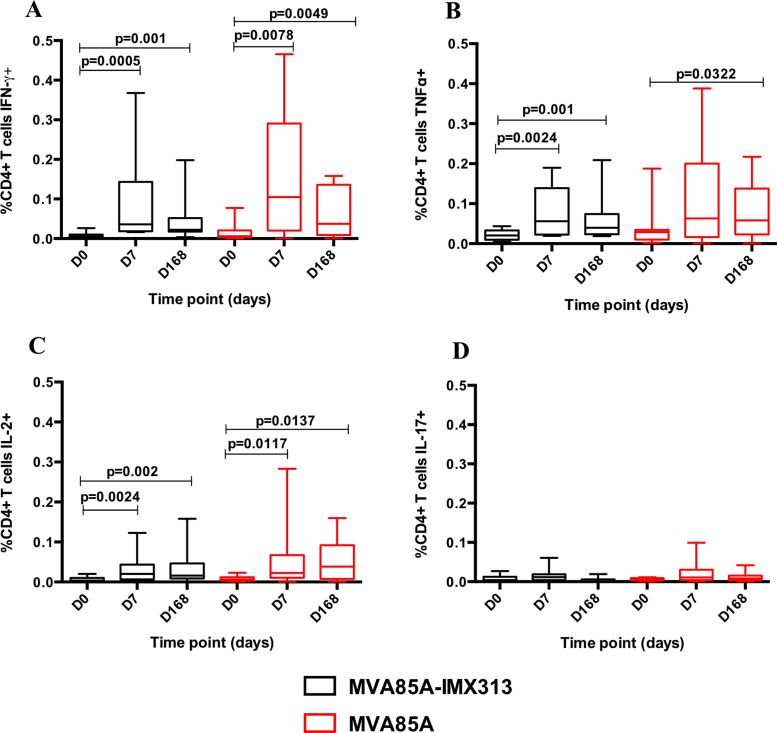
Whole blood ICS Ag85A-specific responses in volunteers vaccinated with MVA85A-IMX313 or MVA85A (5 × 10^7^ pfu) at baseline, day 7 and day 168. Percentages of CD4^+^ T cells producing cytokines are shown in figures (A–D). Lines show median responses in each group, whiskers show inter-quartile range. The Wilcoxon matched pairs signed rank test was used to detect differences between time points in the same group and *p*-values have not been adjusted for multiple comparisons. No significant differences in percentages of cells producing IFN-*γ*, TNF-*α*, IL-2 and IL-17 were detected between the two groups (Mann–Whitney test for AUC, *p* = 0.4799, *p* = 0.5987, *p* = 0.5575 and *p* = 0.3241 for CD4+ T cells IFN-*γ*, TNF-*α*, IL-2 and IL-17 respectively).

Intracellular Ag85A-specific IFN-*γ*, TNF-*α*, IL-2 and IL-17 were examined in stimulated whole blood from volunteers in the two study groups B (MVA85A-IMX313) and C (MVA85A) at baseline, D7 and D168 post-vaccination. No significant differences in percentages of cells producing IFN-*γ*, TNF-*α*, IL-2 and IL-17 were detected between the two groups (AUC, *p* = 0.4799, *p* = 0.5987, *p* = 0.5575, *p* = 0.3241, *p* = 0.6943 and *p* = 0.8325 for CD4+ T cells IFN-*γ*, TNF-*α*, IL-2 and IL-17 and CD8+ T cells IFN-*γ* and TNF-*α*, respectively) (Fig. 4).

CD4+ T cell IFN-*γ* and IL-2 significantly increased in both groups at D7 and D168 (Fig. 4A and C). Group B (MVA85A-IMX313) volunteers had significantly higher percentages of CD4+ TNF-*α*+ T cells at D7 and D168 compared to baseline. A significant increase in percentages of CD4+ T cells producing TNF-*α* was detected in group C (MVA85A) at D168 (Fig. 4B). Ag85A-specific IL-17+ CD4+ T cell responses were detectable following vaccination in both study groups (Fig. 4D). CD8+ T cells producing IFN-*γ* and TNF*-α* were detected at very low percentages in both groups B (median D7 responses of 0.006 and 0.005 for CD8+IFN-*γ*+ and CD8+TNF-*α*+ respectively) and C (median D7 responses of 0.003 and 0.0027 for CD8+IFN-*γ*+ and CD8+TNF-*α*+ respectively) (data not shown).

**Fig. 5:**
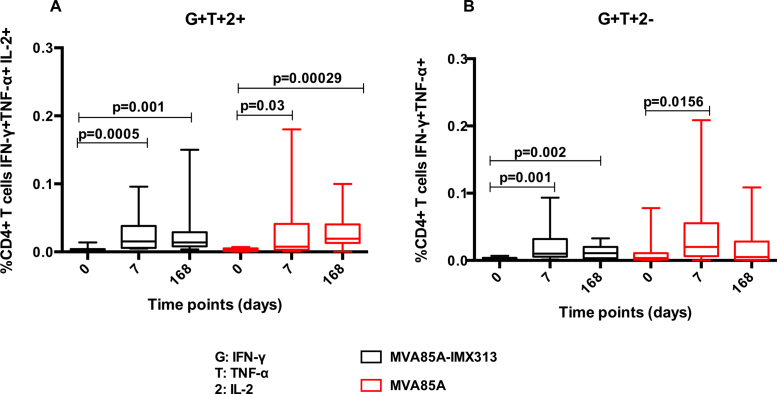
Polyfunctionality of Ag85A-specific CD4+ T cells in volunteers vaccinated with MVA85A-IMX313 or MVA85A (5 × 10^7^ pfu). Percentages of CD4+ T cells simultaneously producing IFN-*γ*, TNF-*α* and IL-2 (A) and those double positive for IFN-*γ* and TNF-*α* are shown in (B). Box and whisker plots show minimum and maximum values with horizontal lines representing medians. The Wilcoxon matched pairs signed rank test was used to detect differences between time points in the same group and *p*-values have not been adjusted for multiple comparisons. No significant differences in percentages of CD4+ T cells simultaneously producing IFN-*γ*, TNF-*α* and IL-2 or double positive for IFN-*γ* and TNF-*α* were detected between the two groups (Mann–Whitney test for AUC, *p* = 0.863 and *p* = 0.518, respectively).

We focussed on the dominant functional phenotypes and found polyfunctional CD4+ T cells, making two or more cytokines simultaneously, cells producing IFN-*γ* in combination with TNF-*α* and IL-2 (Fig. 5A) or double positive for IFN-*γ* and TNF-*α* (Fig. 5B) were all induced following MVA85A-IMX313 or MVA85A vaccination. The magnitude of these responses was not significantly different between the two study groups (AUC *p* = 0.863 and *p* = 0.518 for CD4+ T cells simultaneously producing IFN-*γ*, TNF-*α* or double positive for IFN-*γ* and TNF-*α* respectively (Mann–Whitney)) (Fig. 5). No polyfunctional CD8+ T cells were detected.

#### 3.2.3 Serum IgG responses after MVA85A-IMX313 and MVA85A vaccination

**Fig. 6:**
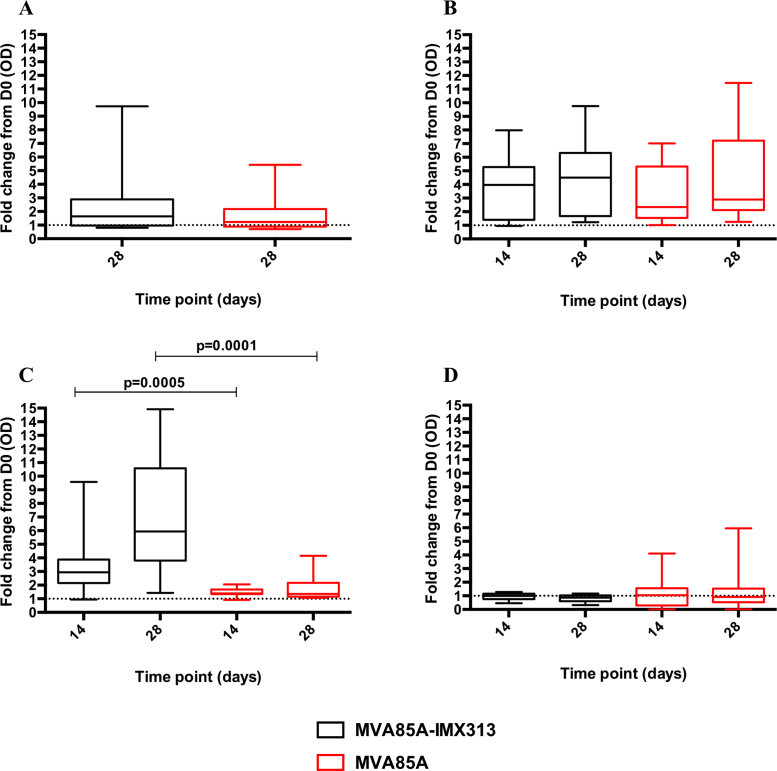
Serum antibody response to Ag85A (A), Wild-type MVA (B), IMX313 (C) and C4bp (D) in the MVA85A and MVA85A-IM313 vaccine groups. Data is presented as fold change responses calculated by dividing each time point's antibody response (measured in optical density) by its corresponding day 0 response. The Mann–Whitney test was used to detect differences between the two study groups.

**Fig. 7:**
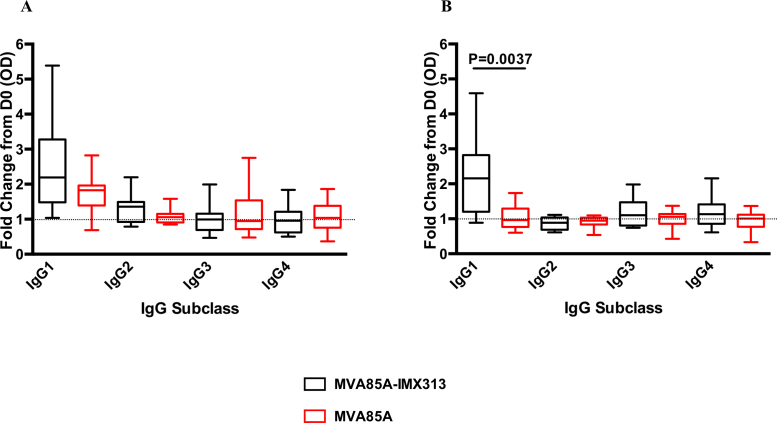
Serum IgG subclass responses to Ag85A (A) and IMX313 (B) in the MVA85A-IMX313 and MVA85A vaccine groups (5 × 10^7^ pfu). Data is measured in optical density after fifty-fold dilution of each serum. Results are presented as fold change responses calculated by dividing each time point's antibody response (measured in optical density) by its corresponding day 0 response. Unpaired *t* test was used to compare between groups.

Levels of serum IgG were assessed in volunteers in the MVA85A-IMX313 and MVA85A (both at 5 × 10^7^ pfu) study groups. There was an increase in serum Ag85A-IgG at D28 in both groups. MVA85A-IMX313 vaccination induced a median OD 405 nm fold change of 1.640 in group B volunteers and MVA85A vaccination induced a median OD 405 nm fold change of 1.227 in group C volunteers (Fig. 6A). These responses were not different between groups (*p* = 0.343). When individual IgG subclasses were examined, in both groups IgG1 responses to Ag85A increased at D28 post-vaccination in both groups (median OD 405 nm fold change = 2.19 and 1.83 in group B and C, respectively). Ag85A-specific IgG2 increased in the MVA85A-IMX313 group with a median OD 405 nm fold change of 1.355. Other 85A-specific IgG subclasses responses did were not induced (Fig. 7A).

Anti-MVA IgG was detected in both groups at D14 and D28 post-vaccination. Group B median OD 405 nm fold change was 3.966 at D14 and 4.506 at D28 compared to group C median OD 405 nm fold change of 2.347 at D14 and 2.891 at D28. No differences were detected in MVA-specific IgG responses between the two study groups (Fig. 6B).

IMX313-specific IgG responses were detectable in the MVA85A-IMX313 vaccination group (B) at D14 post-vaccination and significantly increased at D28 (*p* = 0.0028). As expected, these responses were not detected in the MVA85A alone group (C) (Fig. 6C). When IgG subclasses were examined, the IMX313-specific IgG responses in the MVA85A-IMX313 Group (B) were mainly IgG1 (Fig. 7B). There were no detectable responses to human C4bp at any time point post-vaccination (Fig. 6D).

## 4 Discussion

In this phase I clinical trial we assessed the safety and adjuvant effect of IMX313, which was delivered for the first time to humans, as MVA85A-IMX313.

We demonstrated that this vaccine was well tolerated. The adverse event profiles of both MVA85A-IMX313 and MVA85A were similar and acceptable. Importantly, there were no documented high temperatures in any volunteers. The only severe systemic AEs considered possibly related to vaccination were in a group B volunteer who developed nausea, fatigue and malaise during a diarrhoeal illness that her family also became unwell with. These AEs were included in the analysis because it was not possible to fully distinguish symptoms that were potentially related to the vaccine from symptoms arising from the volunteer's probably infectious pathology.

In this study we show that both MVA85A and MVA85A-IMX313 significantly induce *mycobacteria*-specific immune responses. At the peak time point 1 week post-vaccination both vaccines induced Ag85A-specific IFN-***γ*** ELISpot responses that were not significantly different. This finding is in contrast with previously published data comparing the same vaccines in animal models, where MVA85A-IMX313 was more immunogenic than MVA85A [11]. This may be due to differences in vaccine regime, dose and/or species. In mice and non-human primates that had not received a prior BCG immunisation, two doses of MVA85A-IMX313 were required to demonstrate a significant enhancement of immune responses over MVA85A [12].

It has previously been shown that anti-vector immunity can interfere with insert-induced responses [16]. We studied vaccine-induced cellular anti-vector immunity and demonstrated that both studied vaccines induced comparable anti-MVA T cell responses.

Intracellular whole blood cytokines were comparable between the two study groups and polyfunctional CD4+ T cells were induced by both vaccines. While there are no defined correlates of protection against TB, work on a *Leishmania major* pre-clinical model has demonstrated the importance of the quality of vaccine-induced T cells as measured by cytokines polyfunctionality in protection [17], [18]. A successful vaccine against TB, might require the induction of potent polyfunctional T cells. In the present study we could detect vaccine-induced CD4+ T cells that can simultaneously make IFN-*γ*, TNF-*α* and IL-2 as well as those double positive for IFN-*γ* and TNF-*α*. However, the role of these cells in protection against *M.tb* remains to be investigated. The functional quality of these vaccine-induced CD4+ T cells was not enhanced by fusion to IMX313.

We detected comparable increased levels of anti-85A IgG in both study groups. These responses were predominantly IgG1, and the fold change IgG1 responses were higher in the MVA85A-IMX313 group than after MVA85A. The role of humoral immunity is increasingly recognised as important in TB vaccine development. It was previously shown that *mycobacteria*-specific human IgG could modulate both cellular and humoral immune responses to mycobacteria [19] [20]. IgG1 was reported to be the predominant antibody Isotype present in sera of TB Patients [21]. Hussein et al. [22] suggested a role of IgG1 in TB by enhancing release of TNF-*α* in active patients. It was suggested that the presence of IgG1 and IgG3 antibodies might enhance bacterial uptake and clearance of pathogen via macrophages Fc receptor [22].

In this trial, both vaccine groups had a comparable increase in serum MVA-specific IgG, while IgG responses to IMX313 increased in the MVA85A-IMX313 group and were not detectable in the MVA85A group. The role of these antibody responses is unclear and needs to be further investigated. No antibody cross-reactivity was detected in any of the study groups to the oligomerisation domain of human C4bp, which is likely due to the limited similarity between the IMX313 and human C4bp [11].

Following the failure of MVA85A to enhance efficacy in BCG-vaccinated South African infants [10], there has been a refocussing of efforts to develop a diverse range of potent candidate TB vaccines. These approaches include the inclusion of technologies to enhance immunogenicity, novel antigen delivery systems that induce different phenotypes of T cells, novel routes of immunisation and the assessment of novel candidate antigens. It is likely that multiple approaches will be required.

## 5 Conclusion

Given the encouraging safety data in this study, future research optimising molecular proteins for further evaluation in recombinant viral vectors is warranted. This research may have the potential to accelerate not only TB vaccine development, but also that of other pathogens of global importance including HIV, influenza, *Staphylococcus aureus* and malaria.

## Financial support

A Wellcome Trust Senior Clinical Research Fellowship (Helen McShane; 095780/Z/11/Z) and a financial contribution from IMAXIO funded this trial. An Aeras grant to Oxford (MCA002) funded the Clinical Research Fellow post. The research was supported by the National Institute for Health Research (NIHR) Oxford Biomedical Research Centre based at Oxford University Hospitals NHS Trust and University of Oxford. The views expressed are those of the authors and not necessarily those of the NHS, the NIHR or the Department of Health.

## Figures and Tables

**Supplementary Material 1:**
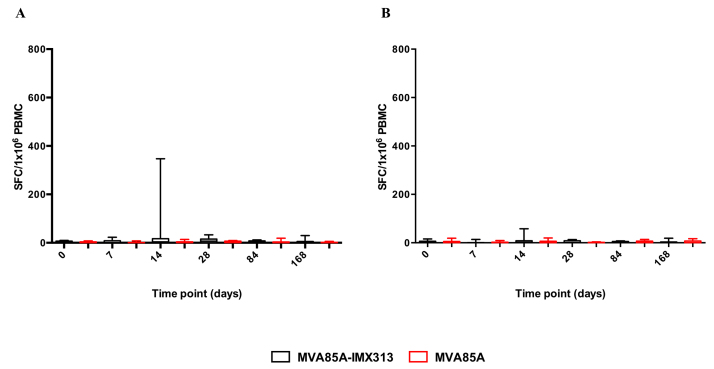
Ex-vivo IFN-γ ELISpot responses to IMX313 (A) and hC4bp (B) in BCG-vaccinated healthy UK adults boosted with either MVA85A-IMX313 or MVA85A. Box and whisker plots show median, inter-quartile range, minimum and maximum values. Stars denote significant changes in responses after vaccination.

**Table 1:** Demographics. Baseline demographics of all study participants by group. Age and time interval since BCG: Means were compared by one-way Anova with Tukey's test for multiple comparisons. No significant difference was observed between groups *p* > 0.05.

	Group A (*n* = 6)	Group B (*n* = 12)	Group C (*n* = 12)
*Age*			
Mean age, years (range)	29.1 (23–42)	34.7 (19–50)	34.7 (20–55)

*Sex*			
Female, *n* (%)	5 (83)	8 (67)	6 (50)

*Place of birth*			
Europe	6	12	12

*Time interval since BCG*			
Mean time, years (range)	17.0 (9–28)	24.4 (12–38)	24.8 (1–42)

**Table 2:** Related adverse events. Shown are the local and systemic adverse events (AE) that occurred following vaccination. Frequency is calculated as the number of volunteers counted once at the time of the highest severity grading of the event.

Symptom and intensity	A (*n* = 6)	B (*n* = 12)	C (*n* = 12)
**Local**			
*Pain*			
Mild	4 (67)	8 (67)	10 (83)
Moderate		1 (8)	
Severe			

*Redness*			
Mild	6 (100)	11 (92)	11 (92)
Moderate		1 (8)	1 (8)
Severe			

*Swelling*			
Mild	5 (83)	11 (92)	11 (92)
Moderate	1 (17)	1 (8)	
Severe			1 (8)

*Warmth*			
Mild	3 (50)	11 (92)	11 (92)
Moderate			1 (8)
Severe			

*Itch*			
Mild	4 (67)	10 (83)	9 (75)
Moderate	1 (17)		1 (8)
Severe			

*Scaling*			
Yes	6 (100)	8 (67)	8 (67)

**Systemic**			
*Feverishness*			
Mild	2 (33)	4 (33)	2 (17)
Moderate		2 (17)	1 (8)
Severe			

*Arthralgia*			
Mild		2 (17)	3 (25)
Moderate			
Severe			

*Myalgia*			
Mild	2 (33)	4 (33)	2 (17)
Moderate			
Severe			

*Fatigue*			
Mild	4 (67)	5 (42)	7 (58)
Moderate	1 (17)		
Severe		1 (8)	

*Headache*			
Mild	3 (50)	5 (42)	3 (25)
Moderate	1 (17)		3 (25)
Severe			

*Nausea*			
Mild	2 (33)	2 (17)	3 (25)
Moderate			
Severe		1 (8)	

*Malaise*			
Mild	4 (67)		2 (17)
Moderate		1 (8)	2 (17)
Severe		1 (8)	

*Other*			
Mild	1^a^ (17)	1^b^ (8)	2^c^ (16)
Moderate			3^d^ (25)
Severe			

a One group A (low dose MVA85A-IMX313) volunteer had 2 mild ‘other’ AEs: they reported feeling ‘disconnected, hazy and sluggish’ on D2, and having ‘non-specific dizziness’ from D0 to D2.

b One group B (MVA85A-IMX313) volunteer had 2 mild ‘other’ AEs: they reported cold symptoms from D6-10 and an ‘upset tummy’ on D1.

c 2 group C (MVA85A) volunteers had mild ‘other’ AEs. One volunteer reported ipsilateral axillary tenderness from D1 to D11, another volunteer reported lightheadedness on the day of vaccination and ipsilateral axillary tenderness on D4-6.

d 3 group C (MVA85A) volunteers had moderate ‘other’ AEs. One volunteer had a mildly raised alanine transferase at screening and D7 post-vaccination, which was moderately elevated at D28, mildly elevated at D84 and normalised by D168 post-vaccination. Another volunteer reported cold/flu symptoms from D1 to D6. Another volunteer reported ipsilateral axillary tenderness from D2 to D17.

## Appendix A Supplementary data

Supplementary Methods 1 Full inclusion and exclusion criteria.
