# Supplementary material for: A first-in-human phase 1 trial to evaluate the safety and immunogenicity of the candidate tuberculosis vaccine MVA85A-IMX313, administered to BCG-vaccinated adults
Source: Vaccine. 2016 Mar 8;34(11):1412–21. doi: 10.1016/j.vaccine.2016.01.062 (PMC4786162; doi:10.1016/j.vaccine.2016.01.062)
Supplement: Supplementary Methods 1 — Full inclusion and exclusion criteria. [file mmc1.docx]

***Inclusion Criteria***

Subjects must meet all of the following criteria to enter the trial:

• Healthy adult aged 18-55 years

• Resident in or near Oxford (for CCVTM) or Birmingham (for WTCRF) and able to travel to Oxford for vaccination for the duration of the trial period

• No relevant findings in medical history or on physical examination

• Confirmation of prior vaccination with BCG not less than 6 months prior to projected trial vaccination date (by visible BCG scar on examination or written documentation)

• Allow the Investigators to discuss the individual’s medical history with their GP

• Use effective contraception for the duration of the trial period (females only)

• Refrain from blood donation during the trial

• Give written informed consent

• Allow the Investigator to register subject details with a confidential database to prevent concurrent entry into clinical trials

• Able and willing (in the Investigator’s opinion) to comply with all the trial requirements

***Exclusion Criteria***

Subjects must meet none of the following criteria to enter the trial:

• Laboratory evidence at screening of latent *M. tb* infection as indicated by a positive ELISPOT response to ESAT6 or CFP10 antigens^a^

• Clinical, radiological, or laboratory evidence of current active TB disease^b^

• Previous vaccination with candidate vaccine MVA85A or candidate vaccine FP85A or any other recombinant MVA vaccine

• Clinically significant history of skin disorder, allergy, immunodeficiency (including HIV), autoimmune disease, cancer (except BCC or CIS), cardiovascular disease, respiratory disease, gastrointestinal disease, liver disease, renal disease, endocrine disorder, neurological illness, psychiatric disorder, drug or alcohol abuse

• History of serious psychiatric condition

• Concurrent oral or systemic steroid medication or the concurrent use of other immunosuppressive agents

• History of anaphylaxis to vaccination or any allergy likely to be exacerbated by any component of the trial vaccine, including eggs

• Any abnormality of screening blood or urine tests that is deemed to be clinically significant or that may compromise the safety of the subject in the trial^b^

• Positive HBsAg, HCV or HIV antibodies^b^

• Female currently lactating, confirmed pregnancy or intention to become pregnant during trial period

• Use of an investigational medicinal product or non-registered drug, live vaccine, or medical device other than the trial vaccine for 30 days prior to dosing with the trial vaccine, or planned use during the trial period

• Administration of immunoglobulins and/or any blood products within the three months preceding the planned trial vaccination date

• Any other significant disease, disorder, or finding, which, in the opinion of the Investigator, may either put the subject at risk or may influence the result of the trial or may affect the subject’s ability to participate in the trial

^a^Subjects discovered to have evidence of latent *M. tb* infection as defined by a positive ELISPOT test will be referred for a plain chest x ray and reviewed with the TB nurse specialists and considered for chemoprophylaxis. If there is any evidence of active TB disease either on clinical or radiological grounds, further investigation and treatment will be offered under the supervision of a consultant physician in respiratory or infectious diseases.

^b^Subjects who are excluded from the trial because they have been discovered during screening procedures to be suffering from a previously undiagnosed condition thought to require further medical attention will be referred appropriately to their GP or an NHS specialist service for further investigation and treatment.
